# Trends and epidemiological analysis of hepatitis B virus, hepatitis C virus, human immunodeficiency virus, and human T-cell lymphotropic virus among Iranian blood donors: strategies for improving blood safety

**DOI:** 10.1186/s12879-020-05405-9

**Published:** 2020-10-07

**Authors:** Azadeh Omidkhoda, Bahman Razi, Ali Arabkhazaeli, Sedigheh Amini Kafi-Abad

**Affiliations:** 1 Department of Hematology and Blood Banking, School of Allied Medical Sciences, Tehran University of Medical Sciences, Tehran, Iran; 2 Department of Hematology, Faculty of Medical Science, Tarbiat Modares University, Tehran, Iran; 3 Blood Transfusion Research Center, High Institute for Research and Education in Transfusion Medicine, Tehran, Iran

**Keywords:** Blood transfusion, Transfusion-transmitted infections, HIV, Hepatitis C virus, Human T-lymphotropic virus 1/2, Hepatitis B virus

## Abstract

**Background:**

Blood transfusion is associated with potential risks of transfusion-transmitted infections (TTIs). Different strategies are needed to monitor blood safety and screen the donors’ efficacy, such as evaluation of the prevalence and trends of TTIs. This study was conducted to evaluate the prevalence and trends of TTIs, including hepatitis B virus (HBV), hepatitis C virus (HCV), human immunodeficiency virus (HIV), and human T-cell lymphotropic virus (HTLV 1/2), and the impact of the donors’ characteristics such as age, sex, and donor status on the prevalence of TTIs in blood donors in seven large provinces of Iran from 2010 to 2018.

**Methods:**

This study was conducted on the data collected from all blood donations in seven Iranian Blood Transfusion Centers including Ardabil, Alborz, Guilan, West Azarbaijan, North, Razavi, and South Khorasan from April 2010 to March 2018. Demographic characteristics, number of donations, donor status, and screening and confirmatory serological results of all blood donations were collected from Iranian Blood Transfusion Organizations (IBTO) national database. The prevalence and trend of HBV, HCV, HIV, and HTLV 1/2 infections were reported according to the donation year and donor’s characteristics.

**Results:**

The analysis of the prevalence and trend of TTIs in 3,622,860 blood donors showed a significant decreasing trend in first-time and regular donors. Additionally, compared to first- time donors, regular donors made safer blood donations with lower risks of HBV, HIV, HCV and HTLV 1/2 (*P* < 0.0001). Although the prevalence of HTLV 1/2 and HBV was higher in females, TTIs had a significant decreasing trend in males and females. Finally, it was found that the prevalence of HBV and HTLV 1/2 increased with age up to 40–49 years and then decreased thereafter.

**Conclusions:**

The decreasing trends of TTIs in Iranian donors during 9 years may indicate that the various strategies implemented by IBTO have been effective in recent years. Other factors such as a decrease in the prevalence of specific TTIs in the general population might have also contributed to these declines.

## Background

Blood transfusion saves the lives of millions of patients worldwide. However, it is not risk-free and is associated with some life-threating complications that affect its useful applications. Statistically, almost millions of blood units were donated in 2019, and estimations suggest that every blood unit has 1% chance of transfusion-associated infections, including transfusion-transmitted infections (TTIs) [1], which may result in mortality and morbidity. They not only impose major burdens on healthcare systems across the world, but also raise questions on the positive aspects of blood transfusion. Thus, utilization of different strategies and screening for hepatitis B virus (HBV), hepatitis C virus (HCV), human immunodeficiency virus (HIV) and human T-cell lymphotropic virus (HTLV 1/2) (in endemic regions) are crucial to estimate the risk of the transfusion of blood and blood products [2–4].

In Iranian Blood Transfusion Organization (IBTO), screening of blood donations for Hepatitis B surface antigen (HBsAg), HIV antibody (Ab), and HCV Ab has been mandatory since 1974, 1989, and 1996, respectively. A retrospective study of 27,442,124 blood donations in Iran from 2003 to 2017 showed a total of 1063 HIV cases, 82,989 HBV cases, and 22,275 HCV cases during 15 years. Accordingly, the period prevalence (15 years) of these infections was 4, 302, and 81 cases in 100,000 donations for HIV, HBV, and HCV, respectively. Moreover, the largest number of infections was identified in the initial years of the study (2003 to 2006) while the smallest number of infections was seen in 2017 (2.5, 53, and 26 cases in 100,000 donations for HIV, HBV and HCV respectively) [5]. During the past decades, various effective strategies have been proposed as the reasons for the decreasing trend of TTIs including implementation of more restrictive donor selection criteria through application of strict and standard questionnaires, effective physical examination procedures prior to donation, educational programs regarding blood donation, and confidential unit exclusion [6, 7]. In addition to screening for three common TTIs in blood donations, the first screening for HTLV 1/2 was performed in 1995 in three northern provinces of Iran (North, Razavi, and South Khorasan provinces) [8]. To determine the prevalence of HTLV 1/2 in donors from other provinces of Iran, HTLV 1/2 was initially tested in 5.4% of all donors in all provinces except the above three provinces in 2007. According to the results, all blood donations have been tested for HTLV 1/2 in addition to HIV, HBV and HCV in seven provinces including North Khorasan, Razavi Khorasan, South Khorasan, Ardabil, Alborz, Guilan and West Azarbaijan since 2007 (unpublished data). A similar study was carried out in 9.8% of donors in 24 provinces except for the above seven provinces in 2011 and based on the results, policymakers in IBTO decided not to extend HTLV 1/2 screening to blood donations in other provinces. Currently, blood donations are screened for HTLV-1/2 only in 7 out of 31 provinces.

The prevalence of HBV, HIV and HCV infections in the Iranian population is 1.7, 0.023%, and less than 1%, respectively [9]. Additionally, the pooled data of a recent meta-analysis showed that the prevalence of HTLV 1/2 was 2.5% in endemic and 0.07–1.8% in non-endemic regions [10]. Monitoring of the prevalence of TTIs in blood donors is essential for blood supply safety. Therefore, IBTO continuously monitors the trends in the prevalence of TTIs to assess the effectiveness of the risk-reducing strategies and screening system. Along with the aforementioned strategies and screening tests that have determinative roles in blood safety and reduction of TTIs, other factors, such as the donation type and donor’s age and sex have been considered important in many studies. In this regard, the results of an eleven-year retrospective study in Iran showed significant differences in the seroprevalence of HBV and HCV across different age groups. The seroprevalence of HBV increased with age. As for HCV, the highest prevalence was observed in the age group 31–40 years and HIV was more prevalent in donors aged 20–30 years. Furthermore, this study found that the seroprevalence of TTIs decreased with an increase in the education level [9]. These results are in accordance with the findings from other countries including Ethiopia, China, and Turkey [11–13]. Another study reported that the seroprevalence of HTLV1 was higher in female, married, and older blood donors [8]. These findings indicate that recognition of the TTIs pattern in different age, sex, and donation type groups can be a complementary approach for decreasing the prevalence of TTIs. Therefore, this study was conducted to evaluate the prevalence and trends of TTIs, including HBV, HCV, HIV and HTLV1/2, and the impact of donors’ characteristics, such as age, sex, and donor status on the prevalence of TTIs in blood donors in seven large provinces of Iran from 2010 to 2018. It seems that this information helps the policymakers to develop effective strategies to improve blood safety.

## Methods

### Data collection

*All blood donations throughout* the country are *screened for* HIV and hepatitis B and C. However, based on epidemiological evidence, HTLV-1/2 screening of blood donations is only performed in seven (Ardabil, Alborz, Guilan, West Azarbaijan, North Khorasan, Razavi Khorasan, and South Khorasan) out of 31 provinces. This retrospective study was conducted on the data collected from all blood donations in blood transfusion centers in these seven cities from April 2010 to March 2018.

Before donation, physical examination and history taking were performed by a qualified physician. The potential donors who were healthy, aged 18–65 years old, and weighed above 50 kg were qualified for donation. Demographic characteristics (age, gender), number of donations, donor status as defined in our pervious publication [14], and screening and confirmatory serological results of all blood donations were collected from the IBTO national database.

### Screening and confirmatory methods

The donations were initially screened for HBsAg, anti-hepatitis C virus antibody (anti-HCV Ab), HIV antigen/antibody (HIV Ag/Ab), and HTLV 1/2 Ab Table 1. The overall sensitivity of each assay was 100% except for INNOTEST HCV Ab IV with a sensitivity of more than 90%. Moreover, the overall specificity was 99.85–99.99% for HBV, 99.83–99.97% for HIV, 99.77–99.99% for HTLV, and 99.81–99.97% for HCV (except 98.5% for Avicenna HCV Ab). If the result of screening for each TTI was repeatedly reactive, confirmatory tests were applied Table 2**.** Based on the TTI type, confirmed positive donors were notified and invited for post-donation counseling and follow-up. Ultimately, the results of the confirmatory tests were used to estimate the prevalence of TTIs.

**Table 1:** The screening tests (2010–2018)

HBsAg	HIV Ag/Ab	Anti-HCV	Anti-HTLV 1/2
1/ Enzygnost HBsAg 5.0 (Dade Behring)	1/ HIV Ag/Ab (BIO-RAD)	1/ Anti-HCV (Avicenna)	1/HTLV 1/2 Elisa versión (MP Biomrdical)
2/ Enzygnost HBsAg 6.0 (Siemens)	2/ Vironostika HIV Ag/Ab (BIOMERIEUX)	2/ Hepanostika HCV Ultra (Beijing United Biomedical Co)	2/ Eiagen HTLV 1/2 I/II Ab (Adaltis)
3/ Monalisa HBsAg Ultra (BIORAD)	3/ Murex HIV Ag/Ab Combination (Diasorin)	3/ Anti-HCV 3.0 Enhanced Save (Ortho)	3/INNOLIA HTLV 1/2 I/II score (Innogenetics)
4/ Murex HBsAg Version 3.0 (Diasorin)	4/ EIAgen Detect HIV 4 Total Screening Kit (Adaltis)	4/ Murex Anti-HCV (Diasorin)	4/HTLV 1/2 І/II Ab version ULTRA (Diapro)
5/Enzygnost HBs Ag 6.0 HBs Ag II (Roche) (Siemens)	5/HIV Combi PT Genscreen (Roche)	5/ INNOTEST HCV Ab IV (Innogenetic)	
	6/ULTRA HIV-Ag-Ab (BIOmRAD)	6/ EIAgen Anti-HCV (V.4) Ab (Adaltis)	
		7/ Enzygnost Anti-HCV 4 (Siemens)	
		8/ Monolisa Anti-HCV Plus Version 3 (BIORAD)	
		9/Elecsys Anti-HCV II (Roche)	
		10/Monalisa Anti-HCV plus (BIORAD)

**Table 2:** The confirmatory tests (2010–2018)

HBsAg Confirmatory Test	HIV Western Blot	HCV Blot	HTLV 1/2 Blot
1/ Enzygnost HBsAg confirm (Dade-Behring)	1/ HIV BLOT 2.2 (Genelabs)	1/ HCV BLOT 3.0 (Genelabs)	HTLV 1/2 I/II BLOT (MP Diagnostics)
2/ Enzygnost HBsAg Confirm (Siemens)	2/ HIV BLOT 2.2 (MP Diagnostics)	2/ HCV BLOT 3.0 (MP Diagnostics)	
3/ Monalisa HBsAg Confirm (BIORAD)	3/ Inno-LIA HIV1/2 Score (Innogenetics)	3/ Inno-LIA HCV Score (Innogenetics)	
	4/ Inno-LIA HIV1/2 Score (Fujirebio)	4/ Inno-LIA HCV Score (Fujirebio)	

### Statistical analysis

The prevalence of TTIs is presented in 100,000 (10^5^) donors in tables and figures according to sex, age, and type of donation. The effect of the donors’ characteristics on the prevalence of TTIs was estimated using relative χ2 (chi-square value/degree of freedom) and 95% confidence interval was calculated. *P* values less than 0.05 were considered significant. Statistical analysis was performed using SPSS v.20.0 (IBM Corp., Armonk, NY, USA). For the trend analysis, a linear trend model was applied using the R statistical software to express the prevalence of TTI as a linear function of time. The “r” values as the correlation coefficients and *P* values are presented in each figure.

### Ethics approval

In IBTO, each donor has to sign a *consent* before *donation. Based on these forms, donors* permit the IBTO to store their information in the blood donor database. This study used the data stored in this national database. The protocol of the study was approved by the Research Committee of High Institute for Research and Education in Transfusion Medicine.

## Results

### Baseline characteristics of donors

From April 2010 to March 2018, 3,622,860 blood donations were made in seven centers, which comprised 14.9% of all blood donations in the country. A marked increase (20.4%) was seen in the number of blood donors over time from 354,695 donors in2010 to 445,345 donors in 2018.

Of all donors, 3,436,921 (94.9%) were male and 185,939 (5.1%) were female. As for the age group, the largest (36.9%) and smallest (15.5%) age group was 30–39 and > 50 years old, respectively. Additionally, 22.4% of donors were < 29 years old and 25.5% were 40–49 years. The majority of the donors (76.7%) were regular donors and 23.3% were first-time donors. The baseline characteristics of the donors are summarized in Table 3**.**

**Table 3:** Characteristics of blood donors in seven provinces of Iran, 2010–2018

Year	Donation Number	Number (Percentage)							
		Sex		Age) year)				Donor status	
		Male	Female	< 29	30–39	40–49	> 50	First time	Regular
2010	354,695	**332,980** (93.9)	**21,715** (6.1)	**79,452** (22.4)	**120,597** (34.2)	**91,511** (25.8)	**63,135** (17.8)	**120,950** (34.1)	**233,744** (65.9)
2011	351,769	**330,232** (93.8)	**21,537** (6.2)	**86,887** (24.7)	**128,396** (36.5)	**88,645** (25.2)	**47,841** (13.6)	**104,124** (29.6)	**247,645** (70.4)
2012	393,317	**371,205** (94.4)	**22,112** (5.6)	**98,330** (25)	**134,514** (34.2)	**98,329** (24.9)	**62,144** (15.8)	**107,769** (27.4)	**285,548** (72.6)
2013	391,210	**371,545** (95)	**19,665** (5)	**91,934** (23.5)	**138,488** (35.4)	**99,759** (25.5)	**61,029** (15.6)	**100,150** (25.6)	**291,060** (74.4)
2014	405,133	**385,176** (95.1)	**19,957** (4.9)	**95,611** (23.6)	**148,279** (36.6)	**98,447** (24.3)	**62,796** (15.5)	**93,991** (23.2)	**311,142** (76.8)
2015	429,629	**410,535** (95.6)	**19,094** (4.4)	**97,526** (22.7)	**167,985** (39.1)	**105,259** (24.5)	**58,859** (13.7)	**92,800** (21.6)	**336,829** (78.4)
2016	419,665	**400,062** (95.3)	**19,603** (4.7)	**102,398** (24.4)	**155,696** (37.1)	**104,916** (25.1)	**56,655** (13.5)	**87,710** (20.9)	**331,955** (79.1)
2017	432,097	**410,844** (95.1)	**21,253** (4.9)	**77,346** (17.9)	**163,332** (37.9)	**116,666** (27)	**74,753** (17.3)	**74,321** (17.3)	**357,776** (82.8)
2018	445,345	**424,342** (95.3)	**21,003** (4.7)	**83,280** (18.7)	**167,895** (37.8)	**120,243** (27)	**73,927** (16.6)	**62,794** (14.1)	**382,551** (85.9)
Total	3,622,860	**3,436,921** (94.9)	**185,939** (5.1)	**815,144** (22.5)	**1,322,344** (36.5)	**923,829** (25.5)	**561,543** (15.5)	**844,126** (23.3)	**2,778,734** (76.7)

### Prevalence and trend of TTIs in blood donors according to donor status

**Fig. 1:**
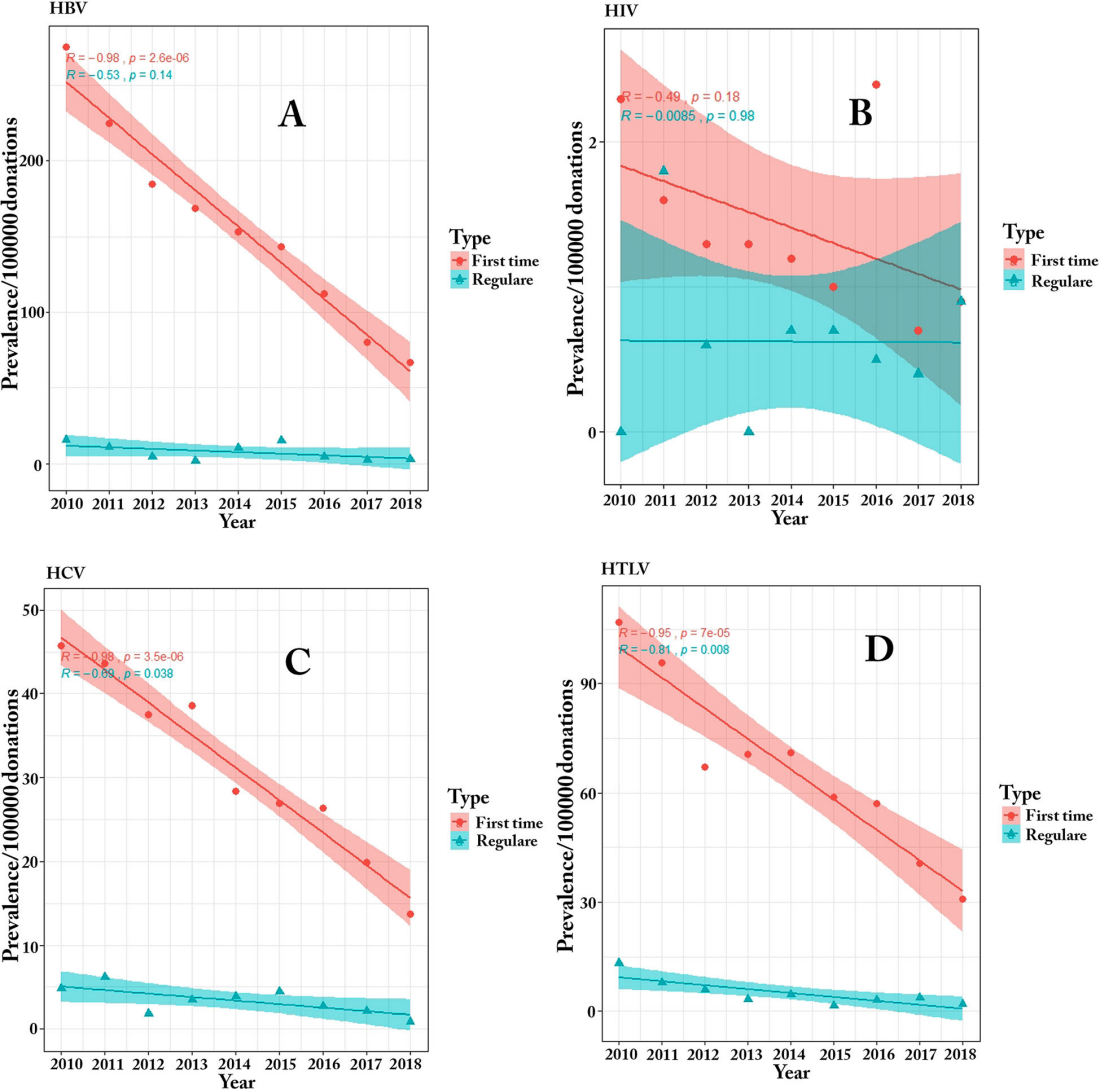
Trend of HBV (**a**), HIV (**b**), HCV (**c**) and HTLV 1/2 (**d**) prevalence in blood donors between 2010 and 2018 by donor status

The analysis of first-time donors (who comprised 23.3% of all donors) showed a decreasing trend in the prevalence of HBV (*P* < 0.0001, R: -0.98), HIV (*P* < 0.18, R: -0.49), HCV (*P* < 0.0001, R: − 0.98) and HTLV 1/2 (*P* < 0.0001, R: − 0.95) from 2010 to 2018. Moreover, the prevalence of TTIs had a similar pattern in regular and first-time donors and there was a marked decline in the prevalence of HBV (*P* = 0.14, R: -0.53), HIV (*P* = 0.98, R: -0.008), HCV (*P* = 0.038, R: − 0.69) and HTLV 1/2 (*P* = 0.008, R: − 0.81) in regular donors. The effect of the donor status on the prevalence of HBV, HCV, HIV and HTLV 1/2 infections by donation year is shown in Fig. 1 and Table 4**.**

**Table 4:** Impact of donor statue on prevalence of TTIs by year

Year	Donation Number	Number of confirmed positive samples Prevalence /100000 donation (95%CI)							
		First time				Regular			
		HBV	HIV	HCV	HTLV	HBV	HIV	HCV	HTLV
2010	354,695	**975,**275 (240–310)	**8** 2.3 (1.5–3.4)	**162** 45.7 (41.6–50)	**379,**106.9 (100.6–1134)	**56** 15.8 (13.5–18.5)	**0**	**17** 4.8 (3.6–6.4)	**47** 13.3 (11.2–15.8)
2011	351,769	**840,**224.9 (215.8–234.4)	**6** 1.6 (0.9–2.6)	**163** 43.6 (39.6–47.8)	**35**8 95.7 (89.8–101.9)	**41** 11.7 (9.7–14.2)	**7** 2 (1.3–3)	**23** 6.5 (5.1–8.3)	**29** 8.2 (6.6–10.2)
2012	393,317	**723,**184.4 (176.2–193)	**5** 1.3 (0.75–2.2)	**147** 37.5 (33.8–41.4)	**263** 67.1 (62.2–72.4)	**17** 4.3 (3.2–5.8)	**2** 0.5 (0.2–1.2)	**7** 1.8 (1.1–2.8)	**23** 5.8 (4.5–7.5)
2013	391,210	**659,**168.5 (160.6–176.7)	**5** 1.3 (0.75–2.2)	**151** 38.6 (34.9–42.6)	**276** 70.6 (65.5–76)	**8** 2 (1.3–3)	**0**	**14** 3.6 (2.6–5)	**13** 3.3 (2.3–4.6)
2014	405,133	**630,**153.1 (145.6–160.9)	**5** 1.2 (0.7–2)	**117** 28.4 (25.2–31.9)	**292** 71 (65.9–76.4)	**42** 10.4 (8.6–12.6)	**3** 0.7 (0.3–1.4)	**16** 3.9 (2.8–5.3)	**19** 4.7 (3.5–6.2)
2015	429,629	**593,**143.6 (136.3–156.2)	**4** 1 (0.54–1.8)	**111** 26.9 (23.8–30.3)	**242** 58.8 (54.2–63.7)	**62** 14.4 (12.7–17)	**3** 0.7 (0.3–1.4)	**18** 4.2 (3.1–5.7)	**6** 1.4 (0.8–2.3)
2016	419,665	**471,**112.2 (105.8–118.9)	**10** 2.4 (1.6–3.5)	**111** 26.4 (23.4–29.7)	**239** 57 (52.5–61.8)	**19** 4.5 (3.4–6)	**2** 0.5 (0.2–1.2)	**11** 2.6 (1.8–3.8)	**13** 3.1 (2.2–4.4)
2017	432,097	**347** 80.3 (74.9–86)	**3** 0.7 (0.3–1.4)	**86** 19.9 (17.3–22.9)	**176** 40.7 (36.9–44.8)	**11** 2.5 (1.7–3.7)	**2** 0.5 (0.2–1.2)	**9** 2.1 (1.4–3.2)	**16** 3.7 (2.7–5.1)
2018	445,345	**298** 66.9 (62–72.2)	**4** 0.9 (0.4–1.7)	**61** 13.7 (11.6–16.2)	**137** 30.8 (27.5–34.4)	**12** 2.7 (1.8–3.9)	**4** 0.9 (0.4–1.7)	**4** 0.9 (0.4–1.7)	**9** 2 (1.3–3)
Total	**3,622,860**	**5536** 152.8 (145.3–160.6)	**50** 1.4 (0.8–2.3)	**1109** 30.6 (27.3–34.2)	**2362** 65.2 (60.4–70.4)	**268** 7.4 (6–9.3)	**23** 0.6 (0.3–1.3)	**119** 3.3 (2.5–4.6)	**175** 4.8 (3.6–6.4)

### Impact of donors’ baseline characteristics on prevalence of TTIs

**Fig. 2:**
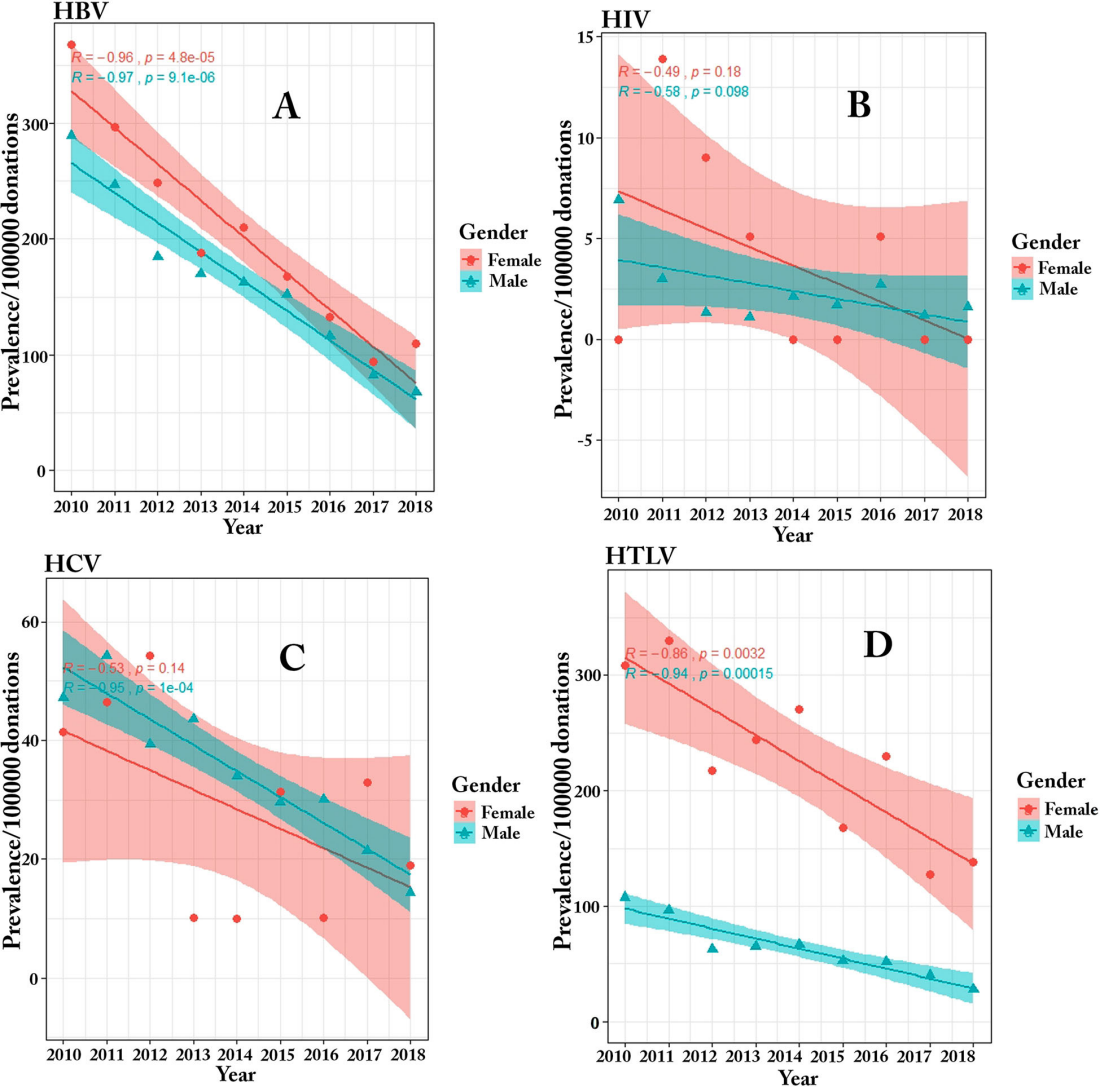
Trend of HBV (**a**), HIV (**b**), HCV (**c**) and HTLV 1/2 (**d**) prevalence in blood donors between 2010 and 2018 by gender

Overall, 94.9 and 5.1% of the donors were male and female, respectively. The results showed a significant decrease in the prevalence of TTIs in males from 2010 to 2018. This significant decrease was also seen in the prevalence of HBV (*P* < 0.0001, R: − 0.96) and HTLV 1/2 (*P* = 0.0032, R: − 0.86) but not for the prevalence of HIV (*P* = 0.18, R: − 0.49) and HCV (*P* = 0.14, R: − 0.53) in females Fig. 2. Moreover, unlike HIV (*P* = 0.2) and HCV (*P* = 0.3), a significant difference was observed in the prevalence of HBV and HTLV 1/2 (*P* = 0.0001) between males and females Table 5.

**Table 5:** Impact of donor gender on prevalence of TTIs by year

	Male					Female				
	**Donation Number**	**Number of confirmed positive samples** **Prevalence /100000 donation (95 CI)**				**Donation** **Number**	**Number of confirmed positive samples** **Prevalence /100000 donation (95 CI)**			
		**HBV**	**HIV**	**HCV**	**HTLV**		**HBV**	**HIV**	**HCV**	**HTLV**
2010	332,980	**963,**289.2 (278.8–299.8)	**23** 6.9 (5.4–8.7)	**157** 47.1 (43–51.5)	**358,**107.5 (102.6–114.1)	21,715	**80,**368.4 (356.7–380.4)	**0**	**9** 41.4 (37.6–45.6)	**67,**308.5 (297.8–319.5)
2011	330,232	**814,**246.5 (236.9–256)	**10** 3 (2.1–4.3)	**179** 54.2 (49.8–58.9)	**318** 96.3 (90.4–102.5)	21,537	**64,**297.2 (286.7–308)	**3** 13.9 (11.7–16.4)	**10** 46.4 (42–51)	**71,**329.7 (318.6–341.1)
2012	371,205	**686,**184.8 (176.5–193.4)	**5** 1.3 (0.75–2.2)	**146** 39.3 (35.6–43.2)	**231** 62.2 (57.5–67.3)	22,112	**55,**248.7 (239.1–258.6)	**2** 9 (7.3–11)	**12** 54.3 (49.9–59)	**48,**217.1 (208.1–226.4)
2013	371,545	**630,**169.6 (161.7–177.8)	**4** 1.1 (0.6–1.9)	**162** 43.6 (39.6–47.8)	**241** 64.9 (60–70)	19,665	**37,**188.2 (179.8–196.8)	**1** 5.1 (3.8–6.7)	**2** 10.2 (8.4–12.4)	**48,**244.1 (234.6–260)
2014	385,176	**626,**162.5 (154.7–170.5)	**8** 2.1 (1.3–3.2)	131 34 (30.5–37.8)	**255** 66.2 (61.3–71.4)	19,957	**42,**210.5 (201.7–219.6)	**0**	**2** 10 (8.2–12.2)	**54,**270.6 (260.6–281)
2015	410,535	**623,**151.8 (144.3–159.6)	**7** 1.7 (1–2.7)	**121** 29.5 (26.3–33)	**215** 52.4 (48.1–57.1)	19,094	**32,**167.6 (160–176)	**0**	**6** 31.4 (28.1–35)	**32,**167.6 (159.8–175.8)
2016	400,062	**464,**116 (96.7–139)	**11** 2.7 (1.8–3.9)	**120** 30 (26.7–33.5)	**207** 51.7 (47.4–56.3)	19,603	**26,**132.6 (126–140)	**1** 5.1 (3.8–6.7)	**2** 10.2 (8.4–12.4)	**45,**229.6 (220.4–239.2)
2017	410,884	**338** 82.3 (76.8–88.1)	**5** 1.2 (0.7–2.1)	**88** 21.4 (18.7–24.5)	**165** 40.2 (36.5–44.3)	21,253	**20** 94.1 (88.3–100.3)	**0**	**7** 32.9 (29.5–36.6)	**27,**127 (120.2–134.2)
2018	424,342	**287** 67.6 (62.7–72.9)	**7** 1.6 (1–2.6)	**61** 14.4 (12.21168–17)	**117** 27.6 (24.5–31)	21,003	**23,**109.5 (103–116)	**0**	**4** 19 (16.5–22)	**29,**138.1 (131–145.6)
Total	3,436,921	**5431** 158 (150–165)	**80** 2.3 (1.5–3.5)	**1168** 33.9 (30.5–37.7)	**2107** 61.3 (56.6–66.3)	185,939	**379,**203.8 (195–212)	**7** 3.8 (2.8–5.2)	**54** 29 (25.8–32.5)	**421,**226.4 (217.3–235.9)

**Fig. 3:**
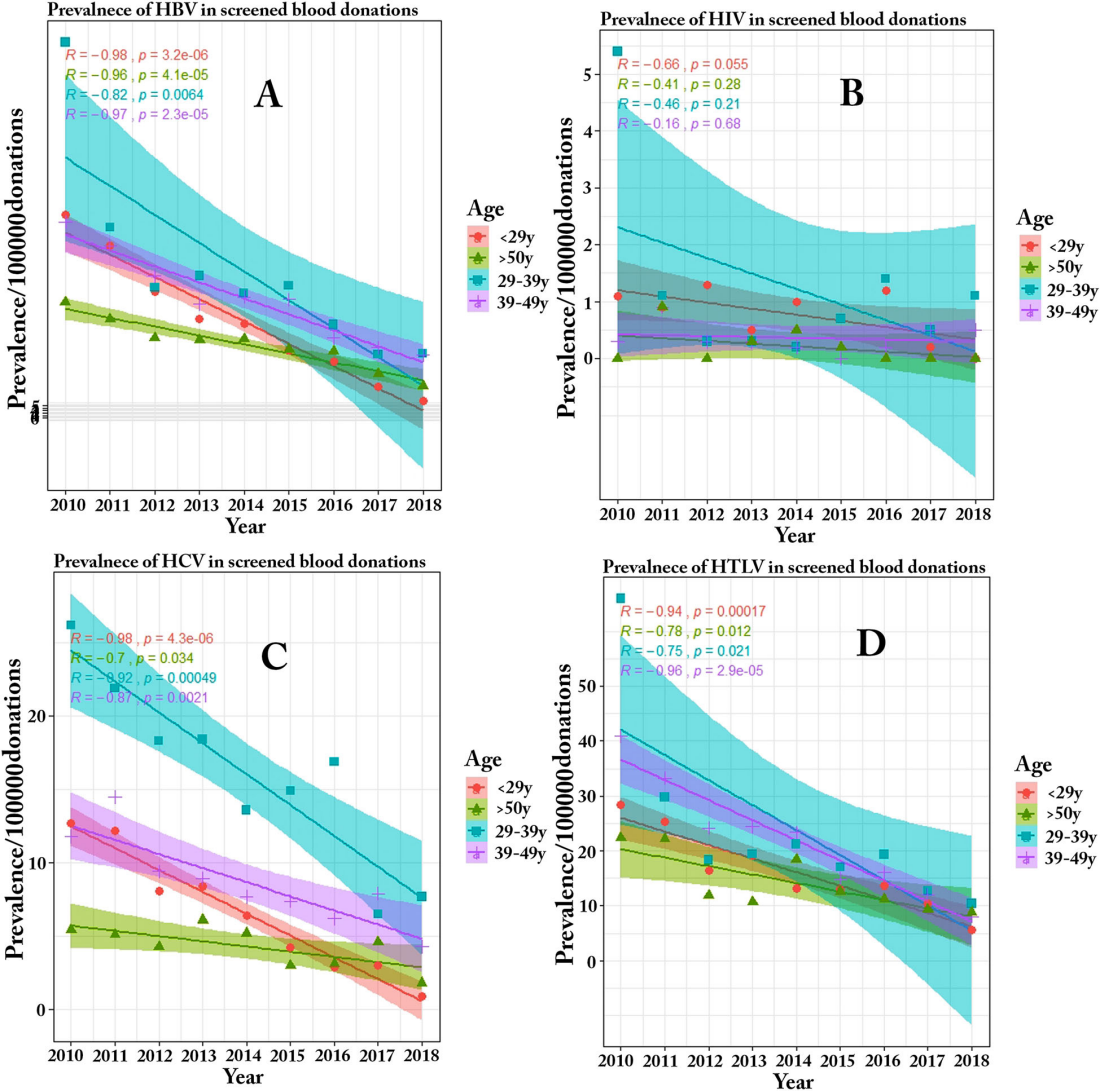
Trend of HBV (**a**), HIV (**b**), HCV (**c**) and HTLV 1/2 (**d**) prevalence in blood donors between 2010 and 2018 by age

Regarding the effect of gender and age on the prevalence of TTIs, a surprising finding was the lower prevalence of HBV and HTLV 1/2 in males compared to females in all age groups. Additionally, the prevalence of HBV and HTLV 1/2 increased with age up to 40–49 years and then decreased thereafter Table 6. Furthermore, regardless of gender, a significant decrease was observed in the prevalence of HBV in all age groups (< 29 years (*P* < 0.0001, R: − 0.98), 29–39 years (*P* = 0.0064, R: − 0.82), 39–49 years (*P* < 0.0001, R: − 0.97), 50 years (*P* < 0.0001, R: -0.96)), HCV (29 years (*P* < 0.0001, R: − 0.98), 29–39 years (*P* = 0.00049, R: − 0.92), 39–49 years (*P* = 0.0021, R: − 0.87), 50 years (*P* = 0.034, R: − 0.7)), and HTLV 1/2 (29 years (*P* = 0.00017, R: − 0.94), 29–39 years (*P* = 0.021, R: − 0.75), 39–49 years (*P* < 0.0001, R: − 0.96), 50 years (*P* = 0.012, R: − 0.78)) from 2010 to 2018. Moreover, the prevalence of HBV and HCV was higher in donors aged 29–39 years compared to other groups, whereas the prevalence of HIV and HTLV 1/2 was higher in donors aged below 29 years and 39–49 years, respectively Fig. 3.

**Table 6:** Impact of donor age on prevalence of TTIs

Male						Female					
Donation Number	**Age** **(Year)**	**Number of confirmed positive samples** Prevalence /100000 donation (95% CI)				**Donation Number**	**Age** **(Year)**	**Number of confirmed positive samples** Prevalence /100000 donation (95% CI)			
		**HBV**	**HIV**	**HCV**	**HTLV 1/2**			**HBV**	**HIV**	**HCV**	**HTLV 1/2**
**3,436,921**	**< 29**	**1236** 36 (32.4–39.9)	**24** 0.7 (0.4–1.4)	**215** 6.3 (4.9–8)	**505** 14.7 (12.5–17.2)	**185,939**	**< 29**	**78** 41.9 (38–46)	**3** 1.6 (1–2.6)	**11** 5.9 (4.5–7.6)	**61** 32.8 (29.4–36.5)
	***P*** **value**	**0.2**	**0.2**	**0.2**	**0.0001**						
	^a^**Chi-square** **Pearson value**	**1.7**	**1.9**	**1.7**	**37.1**						
	**30–39**	**1894** 55.1 (50.6–59.8)	**41** 1.2 (0.7–2.1)	**553** 16.1 (13.7–18.7)	**716** 20.8 (18.2–23.8)		**30–39**	**113** 60.8 (56.1–65.8)	**1** 0.5 (0.2–1.2)	**13** 7 (5.5–8.8)	**112** 60.2 (55.6–65.2)
	***P*** **value**	**0.1**	**0.7**	**0.002**	**0.0001**						
	**Chi-square** **Pearson value**	**1.02**	**0.6**	**9.3**	**119.8**						
	**40–49**	**1538** 44.7 (40.7–49)	**10** 0.3 (0.1–0.8)	**292** 8.5 (6.8–10.5)	**609** 17.7 (15.2–20.5)		**40–49**	**125** 67.2 (62.3–72.4)	**2** 1.1 (0.6–1.9)	1**5** 8.1 (6.5–10)	**158** 85 (79.4–90.9)
	***P*** **value**	**0.0001**	**0.1**	**0.9**	**0.0001**						
	**Chi-square** **Pearson value**	**19.4**	**3.3**	**0.03**	**376.9**						
	**> 50**	**938** 27.3 (24.2–30.7)	**6** 0.2 (0.05–0.7)	**139** 4 (2.9–5.4)	**385** 11.2 (9.7–14)		**> 50**	**88** 47.3 (43.2–51.7)	**1** 0.5 (0.2–1.2)	**14** 7.5 (5.6–9.4)	**116** 62.4 (57.7–67.5)
	***P*** **value**	**0.0001**	**0.3**	**0.0001**	**0.0001**						
	**Chi-square** **Pearson value**	**25.1**	**1.2**	**45.2**	**334.2**						

^a^For each chi-square comparison, the degrees of freedom is 1

## Discussion

This study was conducted to evaluate the prevalence of TTIs, including HBV, HCV, HIV and HTLV 1/2, and the effect of the donors’ characteristics such as age, sex, donor status (first-time and regular) on the prevalence of TTIs in blood donors in seven large provinces of Iran during 9 years from 2010 to 2018.

From an epidemiological point of view, the risk of TTIs has reduced in countries that have applied strict rules for screening of blood donations. Subsequently, the findings of the present study demonstrated a descending trend similar to studies conducted in China [15], the UK [16], and the USA [17]. Additionally, TTIs had a decreasing trend in the first-time donors in 2018 compared to the first-time donors in 2010. These decreasing trends might reflect a decrease in the prevalence of specific TTIs in the general population. In addition, they can be attributed to the implementation of efficient national strategies such as improved public knowledge and awareness about the prevalence and transmission routes of viral infections, especially TTIs, recruiting trained and experienced physicians for donor selection through application of strict and standard questionnaires, implementing donor self-deferral, developing a data registry for blood donors and national donor deferral registry software, educational programs regarding blood donation, using confidential unit exclusion (CUE), pre-donation laboratory screening of first-time blood donors, call back, recall, implementation of well-organized quality systems, good manufacturing practice in blood collection, screening tests, and preparation of blood components, and increased number of regular donors [9, 14, 18].

In the present study, the trend of the prevalence of TTIs according to the donor status revealed a higher prevalence of TTIs in first-time donors compared to regular donors. On the other hand, regular donors made safer blood donations with a lower risk of HCV, HBV, and HTLV 1/2 infections compared to first-time donors, which is in accordance with findings of some previous studies [14, 18, 19]. The high prevalence of TTIs in the first-time donors might be due the fact that a number of first-time donors donate their blood due to reasons such as assessing their health status or benefiting from the effects of blood donation on their health [19]. The lower prevalence of TTIs in regular donors seems to be due to the effect of education on donors, because regular donors are well informed about blood donation procedures [17, 18].

In Iran, the prevalence of HTLV 1/2 in the general population is 2.5% in the endemic regions and 0.07–1.8% in non-endemic regions [10]. As mentioned earlier, the prevalence of HTLV1/2 in blood donors across the country was investigated in two studies in 2007 and 2011 (unpublished data). Based on these results, *HTLV screening* is *commonly performed only* in 7 (Ardabil, Alborz, Guilan, West Azarbaijan, North, Razavi, and South Khorasan) out of 31 provinces. To improve the safety of blood donations and to extend HTLV 1/2 screening to the blood donations in other provinces, we recommended that the risk of different HTLV1/2 screening approaches should be assessed in the country. Based on this risk assessment, the current approach to HTLV1/2 screening may change and first-time donors may be screened for HTLV 1/2 in other provinces instead of screening regular donors for HTLV 1/2 in seven provinces.

An interesting finding of this study was the prevalence of TTIs in male and female donors. In this regard, there was a descending trend in the prevalence of TTIs in both male and female donors during 2010 to 2018, which was similar to other studies across the world. However, a higher prevalence of HBV was seen in females compared to males, which is totally inconsistent with the results of other studies [20–22]. This is while another study [23] reported a higher prevalence of HBV in male donors in Iran (with a narrow margin) compared to female donors during 2008 to 2015. Moreover, the prevalence of HTLV 1/2 was much higher in female donors than in male donors. Satake et al. [24] conducted a study on 1,196,321 blood donors in Japan and found that 3787 donors were positive for anti-HTLV 1/2 Ab and the overall prevalence of infection was 0.66% in males and 1.02% in females. The results of the present study showed an increase in the prevalence of HTLV 1/2 with age in females. In addition, compared to males, blood donation was associated with a higher risk of HTLV 1/2 in all age groups in females, which is consistent with other studies conducted in the same field [25, 26]. A potential explanation for this finding could be the cumulative effects of multiple contacts in lifetime in highly prevalent areas. The higher prevalence of HTLV 1/2 in females is probably due to the higher possibility of male to female transmission during sexual contacts. As shown previously, the frequency of the viral infection in females is twice as high in females as in males [25–27].

The main routes of HTLV 1/2 transmission include mother to child transmission, sexual intercourse, and transfusions from infected blood donors. Collectively, as HTLV 1/2 has a long asymptomatic phase, the risk of transmission by asymptomatic blood donors, particularly in the highly prevalent areas, should be considered and appropriately managed. Implementing strict rules for recruiting safe blood donors and increasing public awareness about HTLV 1/2 infection, especially in highly prevalent regions, might be useful strategies for decreasing TTIs.

The results of the effect of the donor’s age on the prevalence of TTIs revealed that HBV and HCV were more frequent in women of advanced aged compared to their male counterparts. This increase in the prevalence with age may be associated with increased exposure to risk factors over time. Another possible reason for the higher prevalence of HBV in older subjects might be the vaccination plan in Iran. Due to initiation of the HBV vaccination program for all Iranian newborns in 1993 and for teenagers in 2006 [28], the prevalence of HBV is lower in donors aged below 29 years compared to other age groups.

## Conclusion

The decreasing trends of TTIs in Iranian donors during 9 years probably indicate that the various strategies implemented by IBTO have been effective in recent years. Other factors such as a decrease in the prevalence of specific TTIs in the general population might have also contributed to these declines.

## Data Availability

The datasets used and analyzed during the current study are available from the corresponding author on reasonable request.

## Abbreviations

HBV: Hepatitis B virus
HCV: Hepatitis C virus
HIV: Human Immunodeficiency virus
HTLV 1/2: Human T-cell lymphotropic virus
TTIs: Transfusion-transmitted infections
IBTO: Iranian Blood Transfusion Organization
HBsAg: Hepatitis B surface antigen
